# Supplementary material for: Tomographic mapping of the hidden dimension in quasi-particle interference
Source: Nat Commun. 2021 Nov 18;12:6739. doi: 10.1038/s41467-021-27082-1 (PMC8602440; doi:10.1038/s41467-021-27082-1)
Supplement: Supplementary file 1 — Supplementary Information [file 41467_2021_27082_MOESM1_ESM.pdf]

Supplementary Information for 'Tomographic mapping of the hidden dimension in quasi-particle interference'

C. A. Marques,[1,*] M. S. Bahramy,[2,*] C. Trainer,[1] I. Marković,[1,3] M. D. Watson,[1] F. Mazzola,[1] A. Rajan,[1] T. D. Raub,[4] P.D.C. King,[1] and P. Wahl[1]

[1]*SUPA, School of Physics and Astronomy, University of St Andrews, North Haugh, St Andrews, Fife KY16 9SS, United Kingdom*

[2]*Department of Physics and Astronomy, The University of Manchester, Oxford Road, Manchester M13 9PL, United Kingdom*

[3]*Max Planck Institute for Chemical Physics of Solids, Nöthnitzer Strasse 40, 01187 Dresden, Germany*

[4]*School of Earth and Environmental Sciences, University of St Andrews, Irvine Building, St Andrews, KY16 9AL, United Kingdom*

(Dated: November 3, 2021)

* These authors contributed equally.

Supplementary Note 1. SAMPLE CHARACTERISATION

The galena sample used in this study was characterised by Multicollector-Inductively Coupled Plasma Mass Spectrometry (MC-ICPMS) to chemically identify impurities in the system and to determine their respective concentrations. Suppl. Table I shows the part per million of different elements in the sample together with the expected doping.

Supplementary Table I. Summary of MC-ICPMS measurements for different impurity elements. The last column lists the type of doping expected for each impurity.

Element	ppm	2σ	Doping
Bi	3322	100	n-type [1]
Ag	1240	157	p-type[1]
Sb	130	61.9	n-type[1]
Cd	44.9	6	p-type[1]
Tl	18.3	5.3	p-type[1]
Se	194.9	20	isoelectronic
Te	34.6	23.6	isoelectronic

From this analysis, the impurity which is most abundant is Bi which n-dopes the sample, in agreement with our observation from the $\mathrm{d}I/\mathrm{d}V$ spectrum (Fig2 b) that the conduction band is pinned to the Fermi level. As a consequence of the different impurities in the material, in STM topographies we find a large variety of defects coming from these impurity elements. A quantitative comparison of the impurity concentration is difficult because S and Pb vacancies are not captured in the MC-ICPMS analysis.

Supplementary Note 2. STM TOPOGRAPHY

A typical STM topography is shown in Suppl. Fig. 1a, imaged in constant-current mode with a positive bias voltage. The surface is covered with a large diversity of defects, consistent with the natural origin of the crystal. The imaged atomic lattice (inset of Suppl. Fig. 1a) corresponds to the Pb square lattice at the (100) plane. Its periodicity a is the lattice

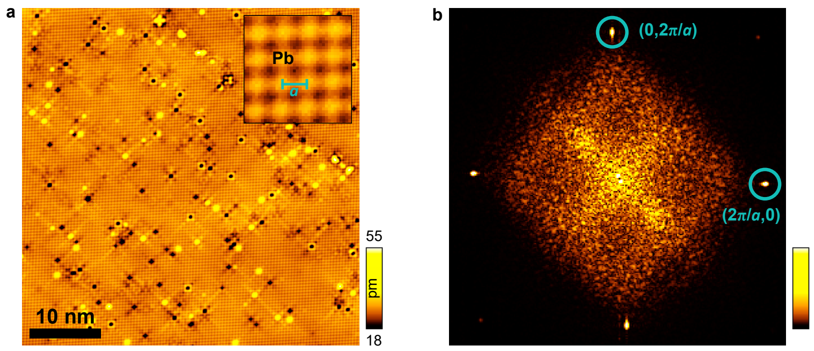

Supplementary Figure 1. **Topography.** **a** Constant-current topography of the surface of PbS at a temperature of 20 K ($V_{\mathrm{set}} = 0.8$ V, $I_{\mathrm{set}} = 0.2$ nA). The inset shows a close up of the imaged atomic lattice, corresponding to the Pb square lattice with lattice constant a. **b** Fourier transformation of **a**, with the atomic peaks highlighted by the circles.

constant of the conventional unit cell. The Fourier transformation of the topography, Suppl. Fig. 1b, shows one set of atomic peaks at $|\mathbf{q}| = 2\pi/a$.

Supplementary Note 3. COMPARISON OF dI/dV AND $d\ln I/d\ln V$

In the dI/dV spectrum, the peak m2 at positive energies appears with a much stronger signal than the other features in the spectrum, as shown in Suppl. Fig. 2a. Its intensity completely dominates, hindering the observation of the other features in the spectra. This is a direct consequence of the exponential bias dependence of the tunneling matrix element. The contribution of the density of states of the sample to the tunneling process at increasing bias voltages becomes exponentially large, dominating the measured signal. In order to observe the band gap and the other peaks described in the main manuscript the energy range was split into three ranges, from 2 to 0.6 V, 0.6 to -0.6 V and from -0.6 to -2 V. They were then measured with different setpoint parameters optimized to observe features in those energy ranges, as shown in Suppl. Fig. 2b. Since they were probed with different setpoint conditions, in order to show the three ranges in the same plot with comparable intensities, we show in Fig. 2b of the main manuscript the Feenstra function instead of dI/dV.

Due to this issue, to study quasiparticle interference, the differential conductance maps were performed with different setpoint conditions, summarized in Suppl. Table II. In order

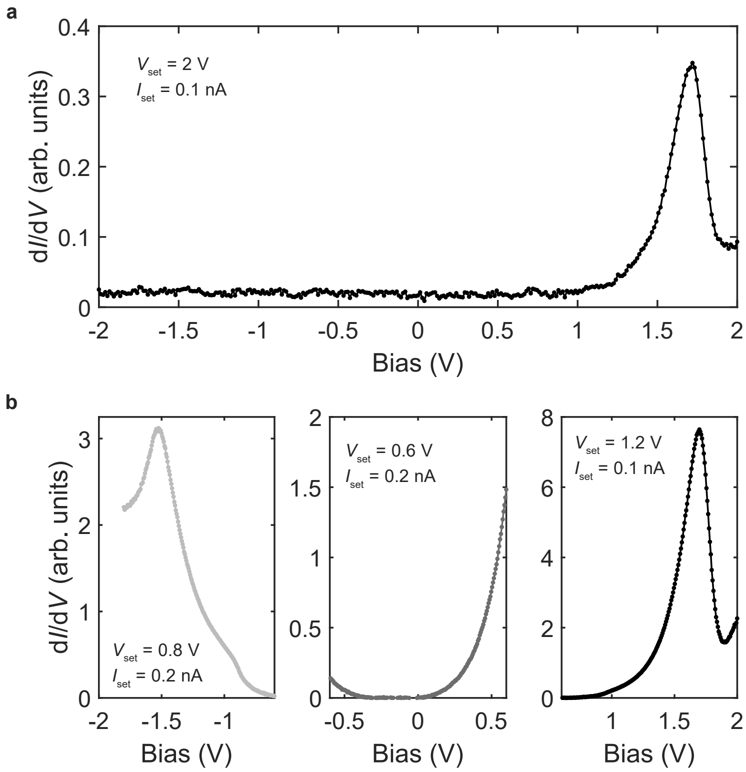

Supplementary Figure 2. **Differential conductance spectra. a** $\mathrm{d}I/\mathrm{d}V$ spectrum in a range of ± 2 V taken with a bias setpoint of 2 V. Only the m2 peak is visible. **b** $\mathrm{d}I/\mathrm{d}V$ spectra for the three ranges shown in Fig.2 b in the main manuscript, taken with different setpoint conditions. They reveal additional features to the peak in **a**, mainly the presence of M2, M1 and m1, as well as the band gap.

to compare the different maps taken, the Feenstra function was calculated and used for the analysis shown in the main manuscript. In Suppl. Fig. 3a and b we show the raw $\mathrm{d}I/\mathrm{d}V$, current and $\mathrm{dln}I/\mathrm{dln}V$ for two energies of $+0.85$V and -0.85V, for both real space and Fourier space.

Supplementary Note 4. PROCESSING OF QPI MAPS

To increase the signal-to-noise ratio, Quasi-particle interference maps in the main manuscript have been symmetrized. The Fourier transform of the $\mathrm{dln}I/\mathrm{dln}V$ maps was first subjected to a drift correction algorithm that uses a geometric transformation to

Supplementary Table II. QPI map parameters for the measurements shown in Fig. 2, 3 and 4 of the main manuscript. ΔE is the voltage range of the map. $V_{\rm set}$ and $I_{\rm set}$ are the setpoint parameters used to regulate the tip height when moving between points of the map, $V_{\rm L}$ is the RMS amplitude of the lock-in modulation, Δx the lateral size of the image and n the number of spectra taken at equidistant points over the length Δx. The maps each cover an area of Δx^2 with n^2 spectra in total.

ΔE (V)	$V_{\rm set}(V)$	$I_{\rm set}$ (pA)	$V_{\rm L}$ (mV)	Δx (nm)	n
0.70 - 1.00	1.00	200	10	38.5	240
1.08 - 1.40	1.40	200	10	37	240
1.20 - 1.60	1.60	300	10	37	240
1.65 - 1.80	1.60	250	10	38.5	240
-0.85 - -1.05	0.80	250	12	37	240
-0.90 - -1.00	0.90	200	10	37	240
-1.10 - -1.60	0.90	200	10	37	240

map the position of the Bragg peaks onto the one expected for a perfect lattice and then symmetrized to impose 4-fold rotational symmetry and mirror symmetry.

Supplementary Note 5. k_z DEPENDENCE OF QPI ENERGY DISPERSION

Suppl. Fig. 5 shows the calculated quasi-particle interference in comparison to the q-vectors extracted from the experimental data, as also shown in Fig. 4a. It becomes clear from the panels that while some k_z planes achieve reasonable agreement (e.g. for $k_z = \pi/c$), some q-vector are only in quantitative agreement between theory and experiment once the correct k_z-plane has been accounted for. For example, while a feature near the quasi-particle peak corresponding to q_1 is visible in Suppl. Fig. 5a, it shows too small a vector in the calculation. The agreement becomes, for this q-vector, much better for $k_z = 0.6\pi/c$ (Suppl. Fig. 5c). Accounting correctly for the contributions of the different k_z planes leads to the excellent agreement shown in fig. 3 of the main manuscript.

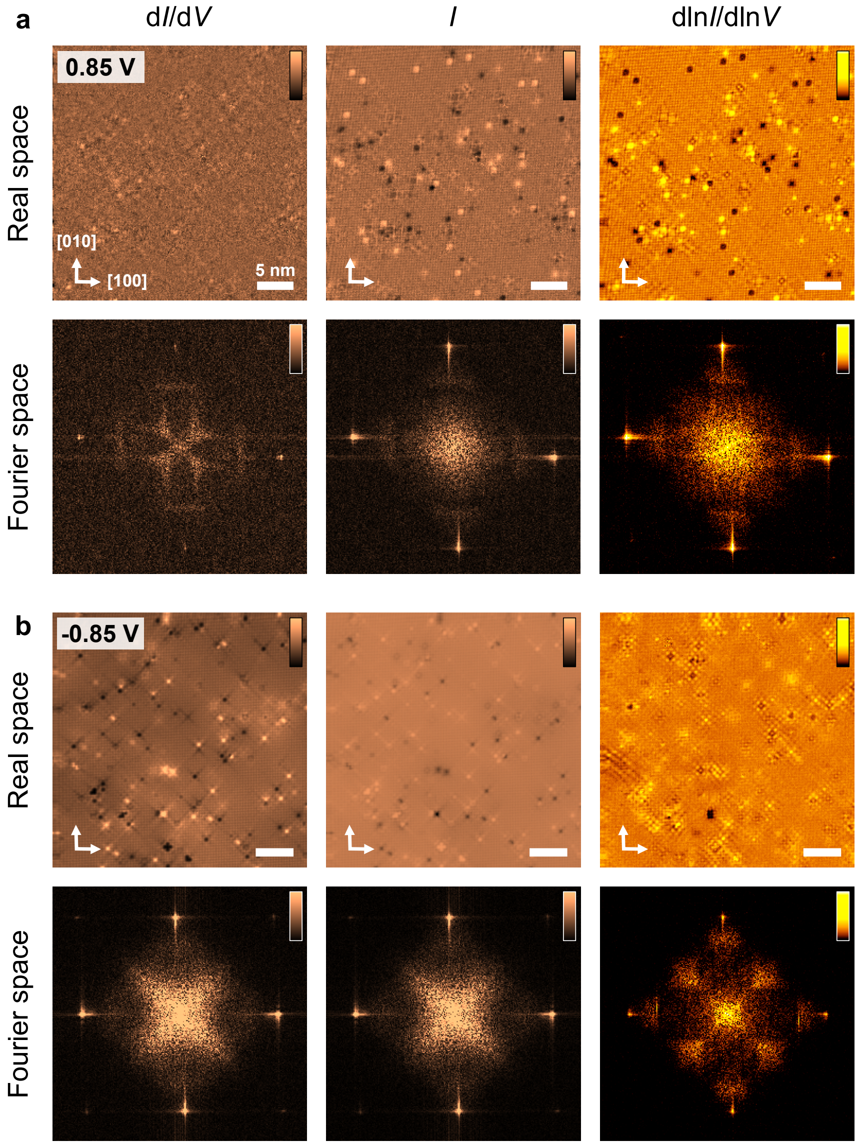

Supplementary Figure 3. **Comparison of conductance maps with Feenstra function.** **a** Top line shows the real space images for $\mathrm{d}I/\mathrm{d}V$, I and $\mathrm{dln}I/\mathrm{dln}V$ respectively, at a bias voltage of 0.85 V. The bottom line shows the correspondent Fourier transform. **b** Top line shows the real space data for an energy of -0.85V and the bottom line the corresponding Fourier transform for $\mathrm{d}I/\mathrm{d}V$, I and $\mathrm{dln}I/\mathrm{dln}V$ respectively.

Supplementary Note 6. BAND STRUCTURE IN THE FOLDED BZ

Suppl. Fig. 6 shows the band structure of PbS on the folded BZ. The maxima/minima M1/m1 do not occur at a high symmetry point of the surface BZ. However, Van Hove

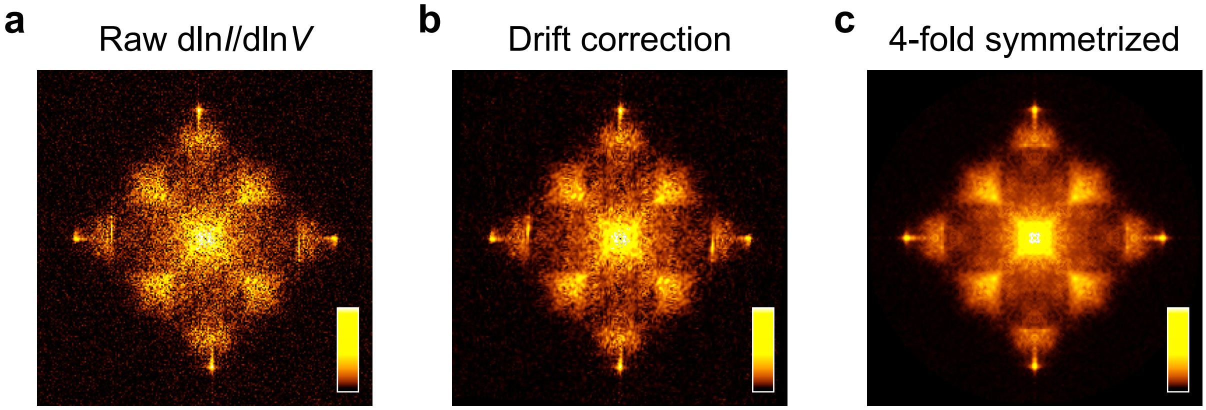

Supplementary Figure 4. **Processing of dln$I$/dln$V$ maps.** **a** Absolute value of the Fourier transform of dln$I$/dln$V$. **b** After drift correction. **c** After symmetrization to increase signal to noise ratio. Energy layer corresponding to -0.85V.

singularities that appeared inside the bulk BZ now appear at the high symmetry point X, for both positive and negative energy states. As a consequence, an additional **q**-vector is observed in the calculations for $k_z > 0.2\pi/c$ which otherwise would not be accounted for.

Supplementary Note 7. DIFFERENCE BETWEEN q_2 AND q_2'

Close to the top of the valence band, the constant energy surfaces of PbS are made up of small pockets centred at the L-points of the FCC BZ, as shown in Suppl. Fig. 7a. The scattering vector q_2 connects the edges of the pockets along the [110] direction at the $k_z = 088\pi/c$ plane, Suppl. Fig. 7b. With decreasing energy, the edges of the pockets stretch along the [110] direction and q_2 decreases in magnitude, Suppl. Fig. 7c. Once the pockets are connected, Suppl. Fig. 7d,e, q_2 becomes zero, and the new scattering vector that dominates along [110] becomes q_2', which connects the flat edges of the tube-like surfaces at the $k_z = 0.88\pi/c$ plane. Moving further away from the Fermi energy, the tube-like surfaces expand, Suppl. Fig. 7f, and the magnitude of q_2' decreases again.

The pockets around the L points connect at the maximum M1 in the band structure (see Suppl. Fig. 6), where the constant energy surfaces change topology from disconnected (Suppl. Fig. 7a) to connected pockets (Suppl. Fig. 7d). As a consequence, there will be two distinct energy dispersions in QPI: a hole-like dispersion that starts with $q_2 \sim (\pi, \pi)$ at the top (bottom) of the valence (conduction) band and becomes zero at the energy of M1,

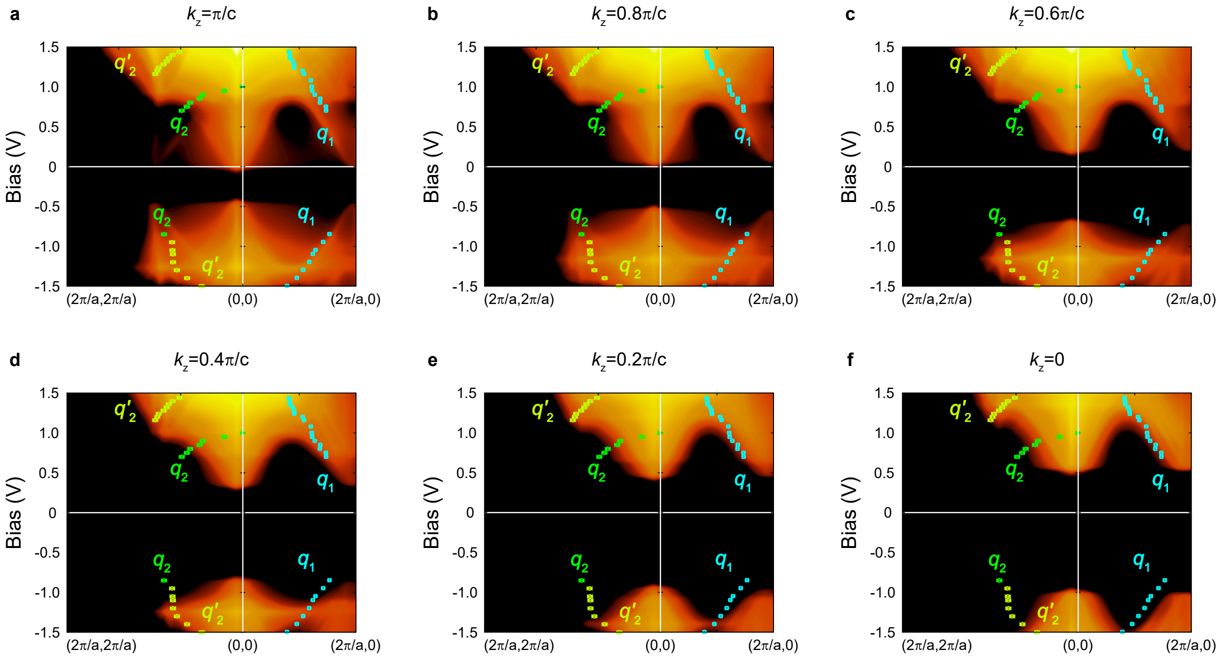

Supplementary Figure 5. **Comparison of the QPI dispersion between model and experiment. a-f** Energy dispersion of the **q**-resolved density of states $\tilde{\rho}(\mathbf{q}, E)$ as shown in Fig. 4d in the main manuscript for decreasing k_z. The peak positions extracted from the experimental data (Fig. 4e) are superimposed on the calculation, the error bars correspond to the **q**-resolution of the dI/dV maps. The panels highlight that the quasi-particle interference cannot be accurately modeled considering only the $k_z = \pi/c$ or $k_z = 0$ planes, but needs to account for the change in the dominant k_z plane.

and a second hole-like dispersion that starts with a finite value at M1, and decreases with increasing absolute energy corresponding to q_2'.

This is clearly seen in energy cuts through both the experimental and theoretical QPI maps at positive energies, Fig. 4d and e of the main manuscript. At negative energies the dispersion of q_2 is different to that at positive energies because additional q-vectors (dotted grey arrow in Suppl. Fig. 7) also appear in QPI as features along the [110] direction, which appear to have a larger contribution at negative energies compared to positive energies. As a consequence, at negative energies, the dispersions of q_2 and q_2' coexist along the same range of energies.

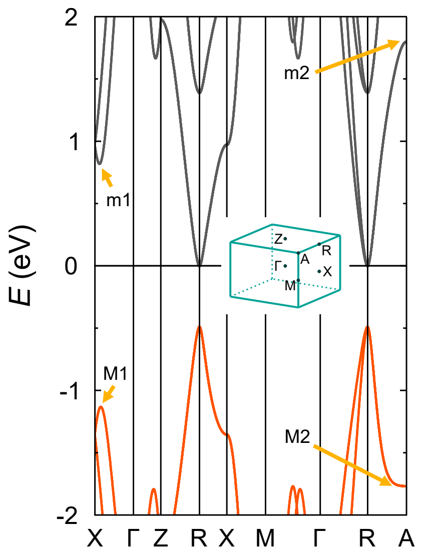

Supplementary Figure 6. **Band structure on the folded BZ.** Band structure of PbS from a tight-binding model considering the BZ of the surface supercell. Van-Hove singularities are indicated by M1 and M2 for the valence band and m1 and m2 for the conduction band, corresponding to the same notation as in the main manuscript. An additional saddle point appears at the X-point as a consequence of the folding.

Supplementary Note 8. ANGLE-RESOLVED PHOTOEMISSION SPECTROSCOPY

We performed angle-resolved photoemission (ARPES) measurements on the same sample (see Methods in the main text), which we can use as a benchmark for the DFT calculation. Fig. 2c of the main manuscript shows the electronic band dispersion along the $\overline{\Gamma}-\overline{\mathrm{M}}$ direction of the surface projected Brillouin zone, aligned with the $\Gamma-\mathrm{X}$ direction of the bulk Brillouin zone (Fig. 2a of the main text) and the $\Gamma-\mathrm{M}$ direction of the folded bulk zone shown in Suppl. Fig. 6. While clear band dispersions are observed, there is substantial spectral broadening. This is a result of the strongly three-dimensional electronic structure of this compound, discussed in the main text. The surface-sensitivity of ARPES means that k_z is no longer a good quantum number, and a Lorentzian distribution in k_z is probed [3]. We estimate the full width at half maximum of this Lorentzian to be 0.4 Å^{-1} from the universal inelastic mean free path [4]. Within a free-electron final-state model, this Lorentzian is centred at $k_z = \frac{\sqrt{2m_e}}{\hbar}\sqrt{E_k \cos\theta + V_0}$, where E_k is the measured kinetic energy, θ is the angle of emission, and V_0 is the so-called inner potential. While this value has not been

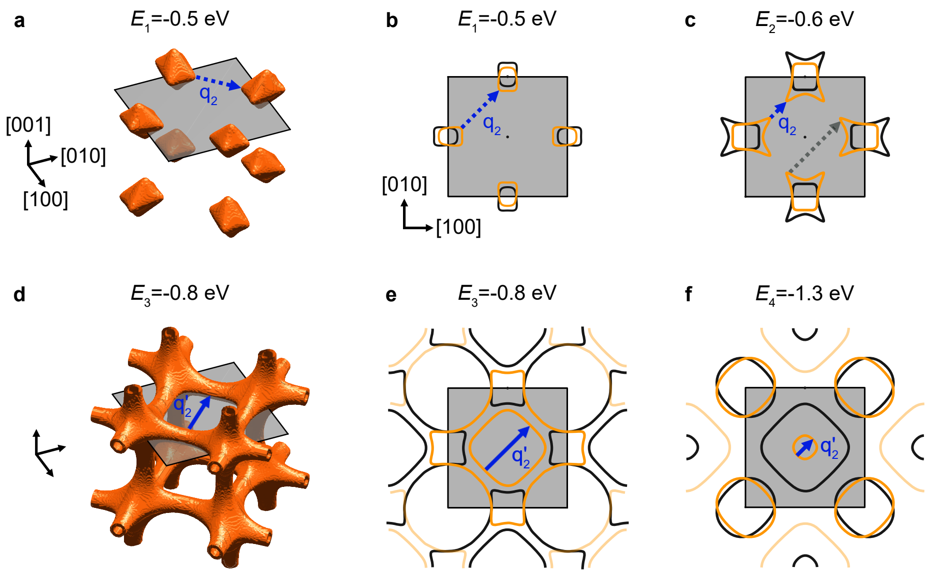

Supplementary Figure 7. **Distinction between** q_2 **and** q_2'**.** **a** Constant energy surface at $E_1 = -0.5$ eV, showing the pockets centered at the L points of the FCC Brillouin zone, based on the tight-binding model from Lent *et al.*[2]. The grey square indicates the $k_z = 0.88\pi/c$ plane and the dotted arrow shows q_2. **b, c** Constant energy cuts at $k_z = 0.88\pi/c$ at $E_1 = -0.5$ eV and $E_2 = -0.6$ eV. The dotted blue arrows indicate q_2, which connects the pockets at the L point along the [110] directions. Additional scattering vectors exist along the [110] direction connecting the flat edges of the stretching pockets, indicated by a dotted grey arrow in **b**. **d** Constant energy surface at $E_3 = -0.8$ eV, where the pockets connect with each other. Once they become connected, q_2 becomes zero and the dominating scattering vector becomes q_2', indicated by the blue arrow, connecting the flat edges of the tube-like surfaces. **e, f** Constant energy cuts at $k_z = 0.88\pi/c$ at $E_3 = -0.8$ eV and $E_4 = -1.3$ eV. The arrow indicates q_2. The edges and sizes of the pockets were manually exaggerated for clarity. The unfolded and folded bands are shown in orange and black, respectively. The square indicates the boundaries of the Brillouin zone of the surface unit cell.

explicitly determined for PbS, we can use a value for the similar compounds, thin-film PbTe (5.9 eV) [5], and bulk SnTe (8.5 eV) [6]. From these values, we estimate that the k_z probed in the experimental dispersion shown in Fig. 2c varies from $k_z \sim 0.3\pi/c$ at the Fermi level to $k_z \sim 0.1\pi/c$ at 5 eV binding energy. We thus compare with a DFT calculation centred at $k_z = 0.2\pi/c$, and where a k_z broadening equal to the experimental k_z resolution has been included. All band features observed in the ARPES measurement are well reproduced by the

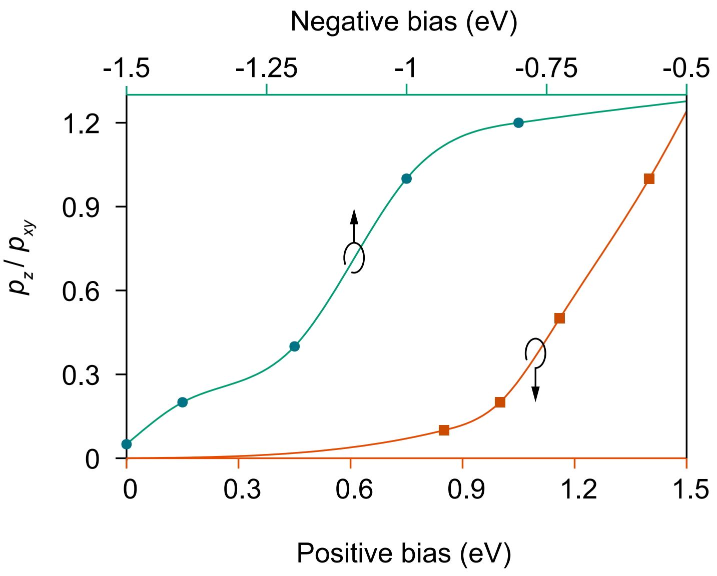

Supplementary Figure 8. **Evolution of orbital contribution to QPI.** Ratio p_z/p_{xy} as a function of energy $E = eV$, used for the QPI calculations in Fig. 4 of the main text. The dots and squares indicate the p_z/p_{xy} values obtained from matching the calculated QPI with that of STM measurements at negative and positive biases, respectively (See Fig. 3 in the main text). The solid lines are the result of a Cspline interpolation smoothly connecting these points.

DFT calculation, expect for some experimental intensity variations due to transition-matrix elements. There is no evidence of surface states in the measured electronic structure. The turning points in the measured low-energy bulk electronic structure are found to be located at slightly lower binding energy in the ARPES than in the DFT, consistent with the STM measurements shown in Fig. 2 of the main text.

Supplementary Note 9. ORBITAL CONTRIBUTION TO QPI

Suppl. Figure 8 shows the expected evolution of the p_z/p_{xy} orbital contribution to QPI for both positve and negative bias potentials.

SUPPLEMENTARY REFERENCES

[1] Jianhua Chen, Lei Wang, Ye Chen, and Jin Guo. A DFT study of the effect of natural impurities on the electronic structure of galena. *International Journal of Mineral Processing*, 98:132 – 136,

2011.

[2] Craig S Lent, Marshall A Bowen, John D Dow, Robert S Allgaier, Otto F Sankey, and Eliza S Ho. Relativistic empirical tight-binding theory of the energy bands of GeTe, SnTe, PbTe, PbSe, PbS, and their alloys. *Superlattices and Microstructures*, 2(5):491–499, jan 1986.

[3] V. N. Strocov. Intrinsic accuracy in 3-dimensional photoemission band mapping. *Journal of Electron Spectroscopy and Related Phenomena*, 130:65–78, 2003.

[4] M. P. Seah and W. A. Dench. Quantitative electron spectroscopy of surfaces: A standard data base for electron inelastic mean free paths in solids. *Surface and Interface Analysis*, 1:2–11, 1979.

[5] Zhenyu Ye, Shengtao Cui, Tianyu Shu, Songsong Ma, Yang Liu, Zhe Sun, Jun-Wei Luo, and Huizhen Wu. Electronic band structure of epitaxial PbTe (111) thin films observed by angle-resolved photoemission spectroscopy. *Physical Review B*, 95:165203, 2017.

[6] Y. Tanaka, Zhi Ren, T. Sato, K. Nakayama, S. Souma, T. Takahashi, Kouji Segawa, and Yoichi Ando. Experimental realization of a topological crystalline insulator in SnTe. *Nature Physics*, 8:800–803, 2012.
